# The Growth of 3T3 Fibroblasts on PHB, PLA and PHB/PLA Blend Films at Different Stages of Their Biodegradation In Vitro

**DOI:** 10.3390/polym13010108

**Published:** 2020-12-29

**Authors:** Vsevolod A. Zhuikov, Elizaveta A. Akoulina, Dariana V. Chesnokova, You Wenhao, Tatiana K. Makhina, Irina V. Demyanova, Yuliya V. Zhuikova, Vera V. Voinova, Nikita V. Belishev, Roman A. Surmenev, Maria A. Surmeneva, Garina A. Bonartseva, Konstantin V. Shaitan, Anton P. Bonartsev

**Affiliations:** 1 Research Center of Biotechnology of the Russian Academy of Sciences, Leninsky Ave, 33, Bld. 2, 119071 Moscow, Russia; vsevolod1905@yandex.ru (V.A.Z.); tat.makhina@gmail.com (T.K.M.); zhuikova.uv@gmail.com (Y.V.Z.); bonar@inbi.ras.ru (G.A.B.); 2 Faculty of Biology, M.V. Lomonosov Moscow State University, Leninskie Gory 1-12, 119234 Moscow, Russia; akoulinaliza@gmail.com (E.A.A.); daryana8@yandex.ru (D.V.C.); veravoinova@mail.ru (V.V.V.); nbelishev@gmail.com (N.V.B.); shaytan49@yandex.ru (K.V.S.); 3 Biological Faculty, Shenzhen MSU-BIT University, No.299, Ruyi Road, Longgang District, Shenzhen 518172, China; tess120@foxmail.com (Y.W.); irinydem@yandex.com (I.V.D.); 4 National Research Tomsk Polytechnic University, Lenin Ave, 30, 634050 Tomsk, Russia; surmenev@tpu.ru (R.A.S.); surmenevamaria@mail.ru (M.A.S.)

**Keywords:** poly(3-hydroxybutyrate), poly-l-lactide, polymer blend, biodegradation, hydrolysis, pancreatic lipase, 3T3 fibroblasts, nanoparticles

## Abstract

Over the past century there was a significant development and extensive application of biodegradable and biocompatible polymers for their biomedical applications. This research investigates the dynamic change in properties of biodegradable polymers: poly(3-hydroxybutyrate (PHB), poly-l-lactide (PLA), and their 50:50 blend (PHB/PLA)) during their hydrolytic non-enzymatic (in phosphate buffered saline (PBS), at pH = 7.4, 37 °C) and enzymatic degradation (in PBS supplemented with 0.25 mg/mL pancreatic lipase). 3T3 fibroblast proliferation on the polymer films experiencing different degradation durations was also studied. Enzymatic degradation significantly accelerated the degradation rate of polymers compared to non-enzymatic hydrolytic degradation, whereas the seeding of 3T3 cells on the polymer films accelerated only the PLA molecular weight loss. Surprisingly, the immiscible nature of PHB/PLA blend (showed by differential scanning calorimetry) led to a slower and more uniform enzymatic degradation in comparison with pure polymers, PHB and PLA, which displayed a two-stage degradation process. PHB/PLA blend also displayed relatively stable cell viability on films upon exposure to degradation of different durations, which was associated with the uneven distribution of cells on polymer films. Thus, the obtained data are of great benefit for designing biodegradable scaffolds based on polymer blends for tissue engineering.

## 1. Introduction

Poly(3-hydroxybutyrate) (PHB) and polylactic acid (PLA) are two most widely used biodegradable and renewable polyesters, which bear the potential to replace petroleum-derived polymers [1]. PHB and PLA have also gained vast attention in the investigation of biomaterials-based products [1,2,3].

**Figure 1:**
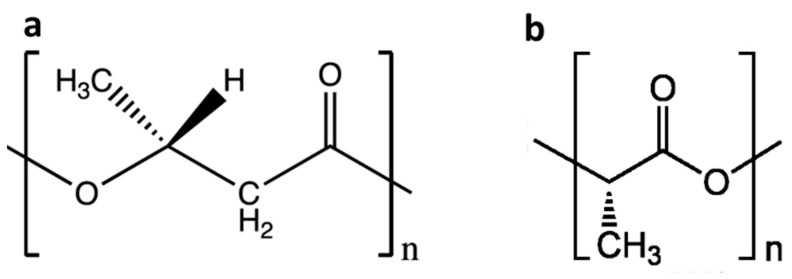
The chemical structure of PHB (**a**) and PLA (**b**).

Poly(3-hydroxybutyrate) (PHB) is a reserve carbon source found in many types of bacteria with chemical structure demonstrated in Figure 1 [4,5]. PHB can be synthesized in various natural environments and it is produced biotechnologically by a series producing strains, such as *Cupriavidus eutrophus* [6] or *Azotobacter chroococcum* [7]. PHB has the ability to degrade into the monomer, 3-hydroxybutyric acid, which is nontoxic and can be removed through metabolism [8,9]. PLA is a polyester produced by chemical synthesis from lactic acid. Lactic acid, the monomer of PLA, is produced from sugar or starch using bacterial fermentation or petrochemical methods. PLA can be also hydrolyzed to its monomer *α*-hydroxy acid within living organisms. This monomer is then further metabolized through tricarboxylic acid cycle [10]. Moreover, PLA degradation products are non-toxic and environmentally friendly, making it a first choice for biomedical and industrial applications, such as biomedical implants and food packaging [11,12,13]. However, the PLA degradation products, which are formed during rapid hydrolysis have no time to be taken up by the organism, which causes a drastic pH decrease in proximity to the implant. Chronic tissue irritation caused by reduced pH is considered a serious problem associated with the use of PLA-based polymer implants [14].

Unlike petro-derived polymers, PHB and PLA exhibit favorable features of biocompatibility, biodegradability, and eco-friendliness, which are very important aspects of biomaterial-based product development. Those features made PLA- and PHB-based materials being exclusively used in many fields ranging from medicine to industrial manufacturing [15,16,17,18,19,20,21]. Commercially available PLA films and packages are becoming indispensable components for daily life products, while biomedical field has witnessed the great potential of PLA-derived polymers as biocompatible sutures and medical implants. PHB plays a crucial role in designing strategies related to regenerative medicine and tissue engineering due to its superior and desirable characteristics. PHB-based biomaterials have demonstrated their validity for production of conventional medical implantation devices, controlled drug delivery systems, and tissues engineered constructs, including repair patches, scaffolds, adhesion barriers, drug delivery platforms and sutures [22,23,24,25]. 

A large number of research has been dedicated to the investigation of these biopolymers’ characteristics, including biodegradability, biocompatibility and mechanical properties, which deepen knowledge of PHB and PLA-based materials [26,27,28,29]. The ability to promote the use of PHAs will be facilitated by the improved understanding of their biodegradability mechanism [30,31]. Despite the presence of a large amount of research that investigates the applications and modifications of PHA, there was a lack of studies regarding the PHA degradation, e.g., surface interaction with cells and dynamic changes of the properties of PHAs. In this research polymer films from PHB and PLA were fabricated for degradation study and cell experiments. 

Currently there is a lack of research investigating the impact of culturing cells on polymer films and scaffolds on polymer degradation [32]. The role of degradation products in cell growth on polymer scaffolds is also poorly understood [33].

The goal of this project is to study dynamic changes of polymer’s properties, and the interaction between biopolymers and cells during the degradation course.

## 2. Materials and Methods

### 2.1. Materials

Bacterial PHB powder was purchased from Biomer (Schwalbach, Germany); PLA and Lipase (isolated from porcine pancreas) were obtained from Merck (former Sigma-Aldrich, Darmstadt, Germany), and bovine serum albumin (BSA) was purchased from Aladdin Industrial Corporation (Shanghai, China).

### 2.2. Fabrication of Polymer Films

Polymer films were prepared by solution-casting method. Chloroform was used as a solvent for both polymers. A solution of chloroform with a polymer (1.2%) was casted into Petri dishes with a diameter of 9 cm, and Petri dishes were left in the fume cupboard for 24 h until all chloroform evaporated. Finally, films were removed from Petri dishes and cut into small disks (diameter: 1.5 ± 0.2 cm, thickness: 40 ± 5 μm, weight: 9 ± 3 mg). A polymer mixture of PLA and PHB (50:50) was made by mixing solutions of PHB and PLA with thorough mixing.

### 2.3. Enzymatic and Non-Enzymatic Hydrolytic Degradation

Non-enzymatic degradation was conducted in phosphate buffer saline (PBS) Merck (former Sigma-Aldrich, Darmstadt, Germany) at pH of 7.4. For enzymatic degradation, lipase was added to PBS with a concentration of 0.25 mg/mL. All solutions were stored in a shaker-incubator (37 °C, 150 rpm). To maintain the buffer quality, free PBS buffer and lipase-containing PBS buffer were changed every 3 days. Films were examined at 4 different degradation points (1 week, 2 weeks, 4 weeks and 6 weeks). To prevent bacterial contribution to the degradation of polymers, sodium azide (2 g/L) was added to the buffer solution.

### 2.4. T3 Cell Seeding Experiments

Murine NIH 3T3 fibroblasts (Biolot, Russia) were used to study the interaction between polymer films and cells at different degradation stages. Cell growth medium was made according to the protocol: Dulbecco’s modified eagle medium (DMEM, PanEco, Moscow, Russia) supplemented with 10% fetal bovine serum (FCS, Biological Industries, Beit-Haemek, Israel), 100 U/mL penicillin (PanEco, Moscow, Russia). The experiment consisted of two parts. For the first part, the experiment was carried out on cell culture plate (24 wells with a diameter of 1.5 cm). Fresh-made films were immersed in ethanol for sterilization (for at least 1 h), followed by washing with sterile PBS. Then, films were flattened and fixed to the bottom of wells. For cell seeding, we added 200 μL of cell suspension (about 2 × 10^5^ cells) and 800 μL of cell growth medium into each well. Finally, the cell culture plate was stored in the CO_2_ incubator (Sony, Minato, Tokyo, Japan) at 37 °C. Degradation of films and the growth of 3T3 cells were observed at 2 different time points: 1 week, 2 weeks. The second cell experiment was performed on a 96-well plate, and the samples included fresh-made films, films after 2-week’s and 6-week-’s degradation. After sterilization, each film was cut into 3 identical pieces, followed by fixation into 96-well plate. For each well, 100 μL of 3T3 cell solution (2 × 10^5^ cells) together with 200 μL cell growth medium was added for cell seeding. The plate was incubated at 37 °C with CO_2_ rate 5.0%, and the cell viability test was conducted at 3 different time points (1d, 3d and 5d). The approvals for all experimental procedures and ethical guidelines were issued by the ISO 10993-1:2009.

### 2.5. Weight Loss Analysis of Polymer Films

Weight loss measurement were carried out at 4 different degradation points (1 week, 2 weeks, 4 weeks and 6 weeks). The changes in the weight of the films during the degradation were determined gravimetrically on the AL-64 scales (Max = 60 g, d = 0.1 mg, Acculab (Sartorius Group), Göttingen, Germany). At each time point, films were taken out of the degradation buffer and washed with distilled water, followed by immersing in 0.1% SDS solution for 24 h. After that, films were washed with distilled water and then dried out. Finally, the weight of each film was measured using microbalance. The weight loss was calculated using the following equation:

(1) $$\mathrm{weight}\;\mathrm{loss}\;\left(\%\right)=\;\frac{W_{0}-W_{d}\;}{W_{0}}\;\times \;100\%$$

where W_0_ is the initial sample weight and W_*d*_ represents measured sample weight after degradation.

### 2.6. Particle Distribution Analysis of Film Degradation Products

Samples of PHB, PLA, and PHB-PLA polymer films of the same weight were washed twice and incubated in milli-Q water at 37 °C with constant stirring. After 7 and 14 days of incubation, the films were removed, and the remaining solutions were centrifuged twice for 5 min at 10,000 rpm. The resulting supernatants were analyzed for polymer degradation products by Dynamic light scattering using a Zetasizer Nano ZS (Malvern Panalytical, Malvern, UK). The distribution of nano- and microparticles size was recorded using Zetasizer Software in four independent replicates. 

### 2.7. Molecular Weight (Mw) Analysis

Ubbelohde viscometer was used to measure the viscosity of PHB, PLA and PHB/PLA blend. Polymer film was dissolved in 7 mL chloroform, and the solution was transferred into viscometer. The experiment was carried out in water bath at 30 °C (Supplementary, Figures S1 and S2). For each sample, the viscosity measurement was repeated 3 times. Molecular weights of PHB and PLA were calculated using Mark-Houwink-Kuhn-Sakurada (MHKS) equation [7]. The molecular weight of PHB/PLA blend was approximated using the known ratios of PHB and PLA and the rate of their decomposition (Supplemental Materials, Figures S1 and S2).

### 2.8. Differential Scanning Calorimetry (DSC)

Thermal properties of PHB, PLA and the blend were measured via differential scanning calorimetry using a DSC 204 F1 Phoenix (Netzsch, Selb, Germany) equipment. The samples were heated from 25 to 200 °C at a heating rate of 10 °C/min in nitrogen environment. The onset and peak temperature of the change in heat capacity was designated as the ^(*onset*)^ and ^(*peak*)^. The accuracy of obtained values did not exceed 1 °C for the temperature measure and 2 J/g for the melting enthalpy. The crystallinity of biopolymer component (Xc) was calculated by the following equation [34]:

(2) $$\mathrm{Xc}\left(\mathrm{PHB}\right)=\;\Delta \mathrm{Hm}/{\Delta H}^{0}m\left(\mathrm{PHB}\right)$$

(3) $$\mathrm{Xc}\left(\mathrm{PLA}\right)=\;\Delta \mathrm{Hm}/{\Delta H}^{0}m\left(\mathrm{PLA}\right)$$

(4) $$\mathrm{Xc}\left(\mathrm{PHB}\right)=\frac{\Delta \mathrm{Hm}}{{\Delta H}^{0}m\left(\mathrm{PHB}\right)}\times \omega \left(\mathrm{PHB}\right)$$

(5) $$\mathrm{Xc}\left(\mathrm{PLA}\right)=\;\frac{\Delta \mathrm{Hm}}{{\Delta H}^{0}m\left(\mathrm{PLA}\right)}\times \omega \left(\mathrm{PLA}\right)$$

where ${\Delta H}^{0}m$ (PHB) and ${\;\Delta H}^{0}$m (PLA)represent the theoretical values for the thermodynamic melting enthalpy of completely crystallized PHB (146.6 J/g) and PLA (93.1 J/g), respectively. ΔHm is the melting enthalpy that was calculated from DSC curve. (PHB) and (PLA) are the weight fractions of PHB and PLA in the blend. All calculations were performed for the second heating cycle. Data was presented as the average of three measurements, *p* values *<* 0.05 were considered statistically significant.

### 2.9. Scanning Electron Microscopy

To investigate the changes of surface structure and cell distribution, scanning electron microscopy (SEM) and fluorescence microscopy were used. For the SEM’s sample preparation standard dehydration process was conducted using different concentrations of ethanol (30%, 50%, 70% and 96%). After that, Hexamethyldisilazane (HMDS) was used to replace ethanol during sample drying. Finally, the specimens were mounted on metal stubs; then stubs were coated with silver in a sputtering device for 15 min at 15 mA (IB-3, Giko, Fukuoka, Japan) and examined under a scanning electron microscope JSM-6380LA (Jeol, Akishima, Tokyo, Japan). For fluorescence microscope films were cut into small pieces, followed by their immersion in PBS buffer containing Calcein for 15 min. After that, samples were removed from solution and washed by PBS. Finally, the samples were analyzed using fluorescence microscope (blue filter) ZEISS Axio Lab A1 (Zeiss, Oberkochen, Germany).

### 2.10. Cell Proliferation Assay XTT Test

In order to determine the viability of NIH 3T3 fibroblasts seeded on films XTT cell proliferation test was used. Films were removed from the medium and cut into identical pieces. The film fragments were then transferred into a 96-well plate. For each well 100 μL of DMEM and 50 μL of reaction solution of Cell Proliferation Kit XTT (Biological Industries, Israel: 2% activation solution plus 98% XTT reagent solution was added followed by the plate incubation for 2 h at 37 °C. Film fragments were taken out of the wells after 2-h incubation. After that, the measurement of the absorbance of the samples was carried out using Zenyth 3100 Microplate Multimode Detector (Anthos Labtec Instruments GmbH, Wals, Austria) at a wavelength of 450 nm; 630 nm wavelength was used as a reference absorbance. Then the calibration plot “cell number—absorbance at 450 nm” was built using the known number of the same cells counted by light microscope with the micrometer scale [35].

### 2.11. Statistical Analysis

Each experiment was conducted from 2 to 3 times with 3 samples for each polymer material (PHB, PLA, PHB/PLA). The non-parametric Kruskal–Wallis test was employed for the statistical evaluation of data using the software package SPSS/PC+ Statistics™ 12.1 (SPSS: An IBM Company, Armonk, NY, USA). The obtained data were represented as mean ± SD (standard error of the mean), and was considered significant for *p* < 0.05.

## 3. Results

### 3.1. Weight Loss of Biopolymer Films

**Figure 2:**
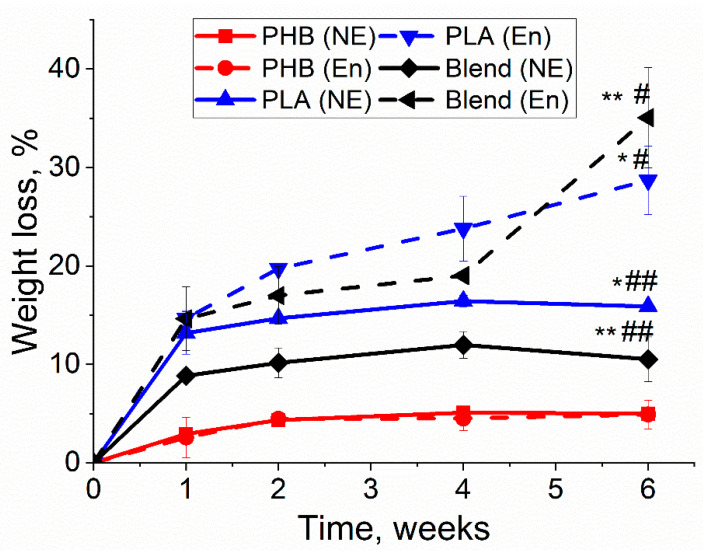
The weight loss of PHB, PLA and PHB/PLA films during enzymatic (En) and non-enzymatic (NE) degradation (NE—incubation in phosphate buffer saline without lipase, En—incubation in phosphate buffer saline with lipase). * PLA (E) vs. PLA (NE), *p* < 0.05; ** Blend (E) vs. Blend (NE), *p* < 0.05; # PLA (E) & Blend (E) vs. PHB (E), *p* < 0.05; ## PLA (NE) & Blend (NE) vs. PHB (NE), *p* < 0.05.

In the degradation experiment, the dynamic changes of the main physicochemical properties concerning three different biopolymers (PLA, PHB and blend 50/50) were measured. Weight loss occurred at every degradation point, though it varied in different polymer films, as shown in Figure 2. PLA and PHB/PLA with enzymatic degradation had a greater weight loss than in conditions of non-enzymatic hydrolytic degradation, whereas both degradation types caused the approximately same weight loss of PHB films. In both enzymatic and non-enzymatic degradations, weight loss increased with the increment of time with access to a relative plateau. Among three types of polymer films, PLA and PHB/PLA blend had greater weight loss values compared with PHB. Notably, PHB/PLA blend exhibited the highest weight loss (up to 35%) after 6 weeks’ enzymatic degradation though it had smaller values than PLA at other observation points.

**Figure 3:**
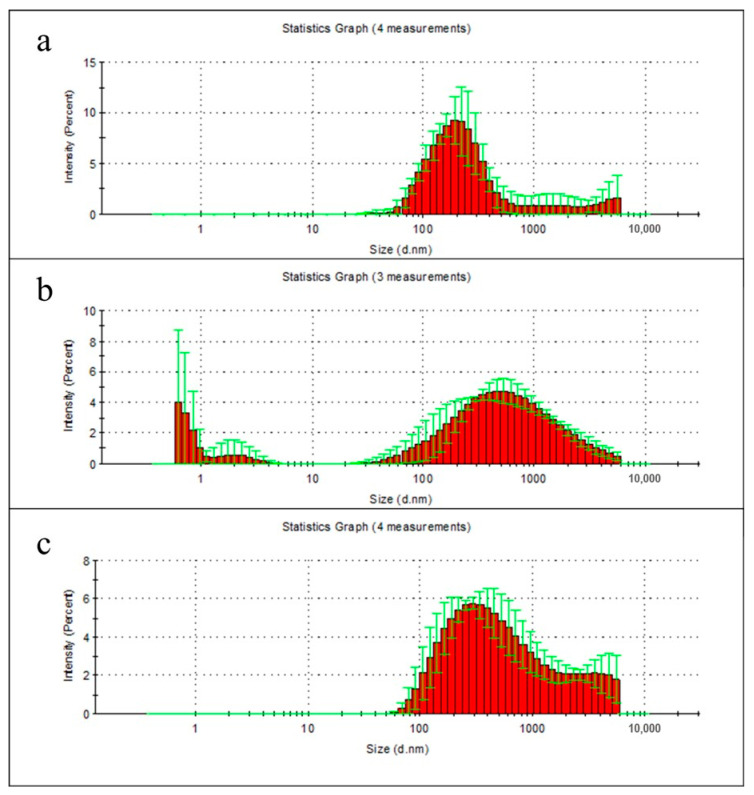
Size distribution of particulate degradation products of PHB (**a**), PLA (**b**), and PHB/PLA blend films (**c**) after 1 week incubation in milli-Q water. Data presented as mean ± SD.

During the first two weeks, the greatest weight loss of all samples was observed, regardless of the lipase presence in the solution. To determine the nature of these changes, the degradation products of polymer films in milli-Q water were examined using dynamic light scattering technique. According to the data from the first week measurements, the particles of polymer films degradation products were hard to detect in milli-Q water, due to their low concentration (especially for PLA) (Figure 3a–c). It should be noted that the low concentration of degradation products can reduce the accuracy of measuring their size. 

However the PHB sample had a one relatively narrow peak at 205 nm, PLA sample had two peaks, whereas the PHB/PLA blend sample had a wide peak with a relatively wide fraction of large particles, which is consistent with the data on the weight loss of polymers in the first week of their non-enzymatic degradation.

**Figure 4:**
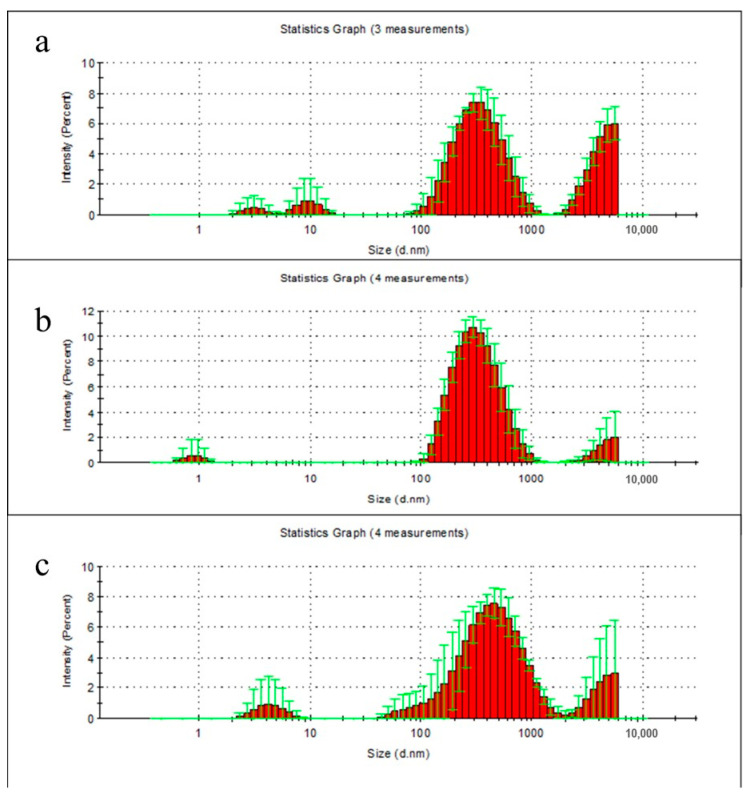
Size distribution of particulate degradation products of PHB (**a**), PLA (**b**), and PHB/PLA blend films (**c**) after 2 weeks incubation in milli-Q water. Data presented as mean ± SD.

At the second week, there were clear peaks for particulate degradation products of PHB with the following maxima: 3, 10, 340 and 5500 nm, PLA: 0.9, 300 and 5500 nm (the last—measured value limit), and for PHB/PLA blend films: 4.1, 340–530 and 5500 nm (the last—measured value limit) (Figure 4a–c).

### 3.2. Molecular Weight Loss of Biopolymer Films

**Figure 5:**
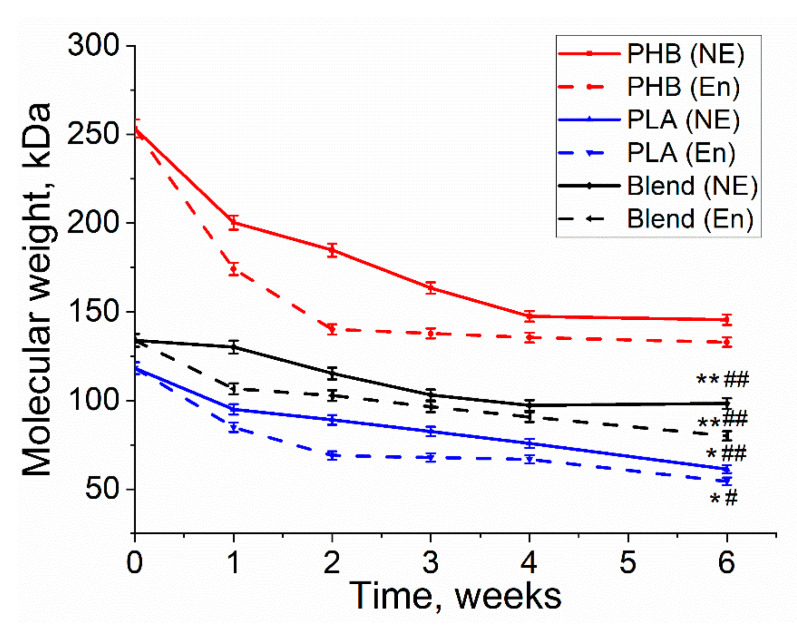
The loss of molecular weight of PHB, PLA, and PHB/PLA during enzymatic (En) and non-enzymatic (NE) degradation. * PLA (E) vs. PLA (NE), *p* < 0.05; ** Blend (E) vs. Blend (NE), *p* < 0.05; # PLA (E) & Blend (E) vs. PHB (E), *p* < 0.05; ## PLA (NE) & Blend (NE) vs. PHB (NE), *p* < 0.05.

Molecular weight is one of the key properties of biopolymers. Its decrease is a good indication of the ongoing degradation process. It can be observed that the molecular weight decreased for all three polymers during both enzymatic and non-enzymatic degradation (Figure 5). In comparison to non-enzymatic hydrolitic degradation, all three polymers had larger molecular weight loss with the involvement of lipase in enzymatic degradation. Moreover, PHB had the highest molecular weight loss (45%) under enzymatic degradation, whereas PLA had smaller molecular weight loss (41%) and the PHB/PLA blend demonstrated the smallest molecular weight loss (36%). It can also be noticed that PHB had the highest initial molecular weight (253 kDa), while PLA and the blend had smaller initial molecular weight values. Despite that the viscosity of PHB had a drastic decrease during the first 3 weeks, no significant changes in the viscosity were observed in the last two weeks of degradation. The decrease in MM in PHB/PLA blend was more even.

**Figure 6:**
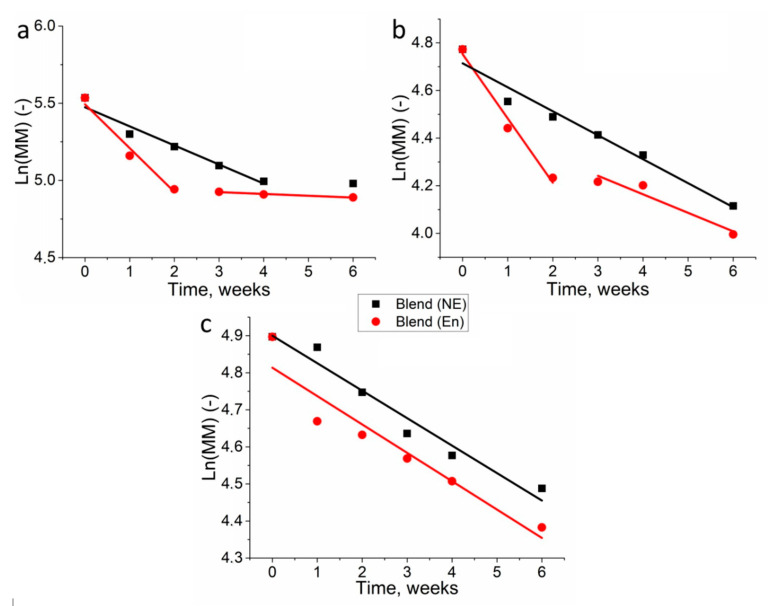
A linearized model of first-order kinetics applied on the degradation of PHB in phosphate buffer without lipase (NE) and phosphate buffer with lipase (E) (**a**), phosphate buffer without lipase and phosphate buffer with lipase (**b**), and PHB/PLA in phosphate buffer without lipase and phosphate buffer with lipase (**c**).

The first-order kinetic model on the observed exponential decrease of molecular weight in time (Figure 6, Table 1) was applied for polymer film degradation in PBS without enzyme and in lipase solution. These values indicated much higher degradation kinetics of the studied polymers than previously published PHB degradation in similar environment [36].

The obtained data indicate different degradation kinetics of homopolymers and their blend. A similar kinetics of degradation was observed for PHB and PLA in lipase solution. In both cases, the greatest decrease in MM was observed during the first two weeks of incubation. In addition, the k_D_ of both polymers was remarkably close. In the following days, the decomposition rate decreased several times. However, in case of the blend of these polymers, the kinetics of degradation was a linear throughout the experiment, both in lipase solution and in phosphate buffer.

The study of the degradation kinetics of various polymers also makes it possible to assess the presence of the autocatalysis process in the decomposition reaction. To evaluate the applicability of the specific model, the curves of degradation were calculated via the statistical correlation coefficients. In order to achieve this, the values 1/MM and ln (MM) were plotted against time, where 1/MM corresponds to a non-catalytic model and ln (MM) corresponds to an autocatalytic one. The correlation coefficients for the respective models are shown in Table 2.

The data in Table 2 suggests that the autocatalysis process takes part in the decomposition of PLA in PBS and in the decomposition of the PHB/PLA blend.

**Figure 7:**
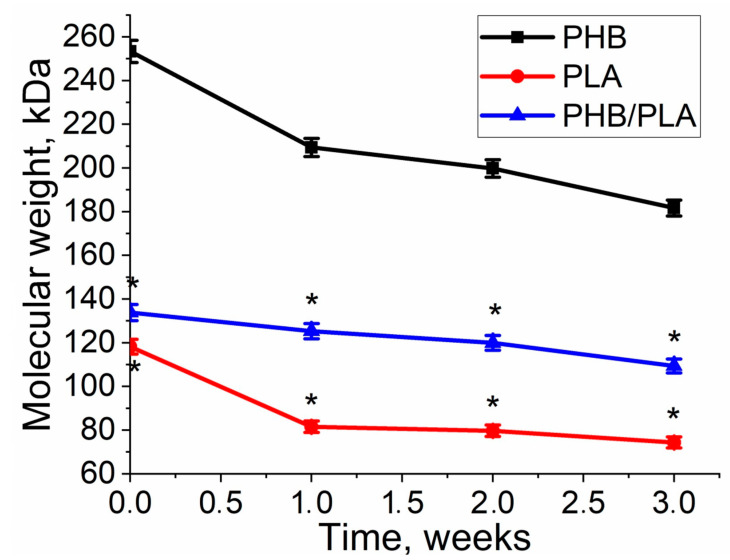
Molecular weight loss of polymers during cultivation of 3T3 fibroblasts on the PHB, PLA, and PHB/PLA blend films. *—PLA & PHB/PLA vs. PHB *p* < 0.05.

To study the influence of seeded cells on polymers’ degradation, the changes in corresponding properties of polymer films during cell culture on them were analyzed. The molecular weight of all polymers decreased during the cell cultivation on the films. As to the experiment, while being in solution, it was observed that the changes in homopolymers occurred nonlinearly. In the first week, there was a stronger decrease in MM in PLA—by 31%, and in PHB—by 17%, versus 6.5% in the PHB/PLA blend (Figure 7).

The application of first-order kinetic model on the observed exponential decrease of molecular weight in time provided the value k_D_ = (0.104 ± 0.02) week^1^ for PHB, (0.1412 ± 0.055) week^1^ for PLA and (0.065 ± 0.064) week^1^ for PHB/PLA blend for degradation during cell experiment. In this case, k_D_ for PHB and PLA biodegradation kinetics was similar, whereas k_D_ of PHB/PLA blend was significantly lower in comparison with two other homopolymers. However, the values of k_D_ for PHB and PLA were closer to values of k_D_ during non-enzymatic hydrolytic degradation than to enzymatic degradation. Noteworthy that k_D_ of PHB/PLA blend was even the same for all experiments. 

The contribution of autocatalysis was also evaluated (Table 2). High correlation coefficients for the non-autocatalytic and autocatalytic models correspond to the polymers PHB and PHB/PLA. This indicates the acceleration of the degradation process by means of autocatalysis. In the degradation of PLA, autocatalysis, in accordance with the coefficients, did not not take place (0.85 for non-autocatalytic and 0.74 for autocatalytic).

### 3.3. Crystallinity Analysis

**Figure 8:**
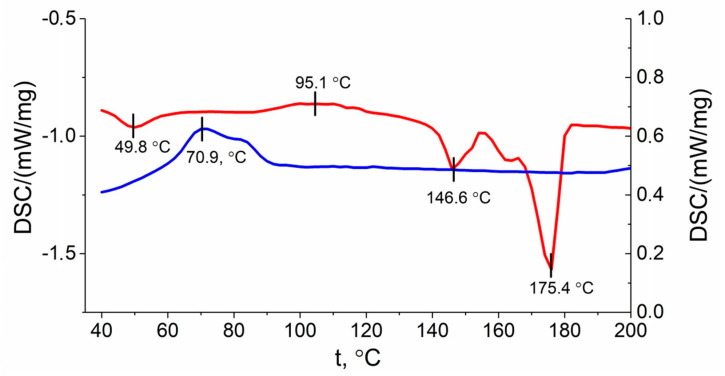
DSC curves of PLA/PHB obtained from second heating run (red line), cooling run (blue line).

Thermal properties of polymers (e.g., crystallinity (X_c_), melting temperature (T_m_)), may encounter changes upon degradation process. The DSC curves of PLA/PHB blend with a heating rate of 10 °C/min is shown in Figure 8. 

Based on the DSC data, upon heating a peak in the glass transition temperature can be seen at 49.8 °C (represented as a red line in Figure 8). The peak of PLA crystallization was detected at 95.1 °C. After that the melting point peaks of PLA and PHB at temperatures of 146.6 °C and 175.4 °C respectively were obtained. During sample cooling, the PHB crystallization peak was observed at 70.9 °C. These results indicate that the crystallinity and crystallization rate of PHB are much higher than PLA [26].

As shown in Table 3, PHB films maintained a relatively stable melting temperature. After 6 weeks PHB crystallinity with non-enzymatic degradation was slightly decreased, while PHB crystallinity after enzymatic degradation was slightly increased. The crystallinity of PLA films dropped from 34% to 29% for both enzymatic and non-enzymatic degradation (Table 3) in spite of the different changes in melting temperature. PHB component in blend had an increase in crystallinity after 6 weeks degradation, whereas the enzymatic degradation showed an increase in crystallinity, as opposed to non-enzymatic degradation. With respect to the PLA component, crystallinity was slightly increased after non-enzymatic degradation, and decreased after enzymatic degradation. Besides, the changes in melting temperature in PHB/PLA blend had a similar pattern with respect to pure PHB and PLA.

### 3.4. Cell Viability Analysis

**Figure 9:**
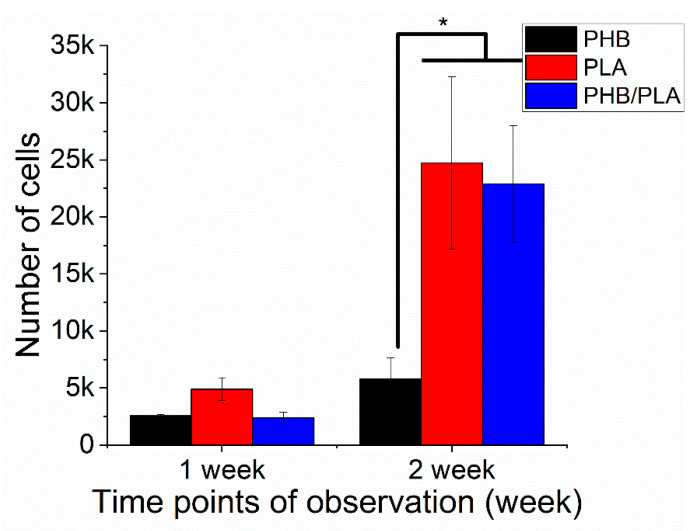
Cell viability test (cell experiment on fresh films). *—*p* < 0.05.

Cell viability is an essential criterion assessing the biocompatibility of polymer films, where high viability value usually indicates better cell proliferation. In the first cell experiment, fresh films were used to evaluate the cell proliferation on the films from the three different polymers. As shown in Figure 9, 3T3 cells proliferated on all polymer films after 2 weeks incubation. However, PLA and PHB/PLA blend had higher cell viability than PHB, suggesting better cell proliferation on PLA and polymer blend films.

**Figure 10:**
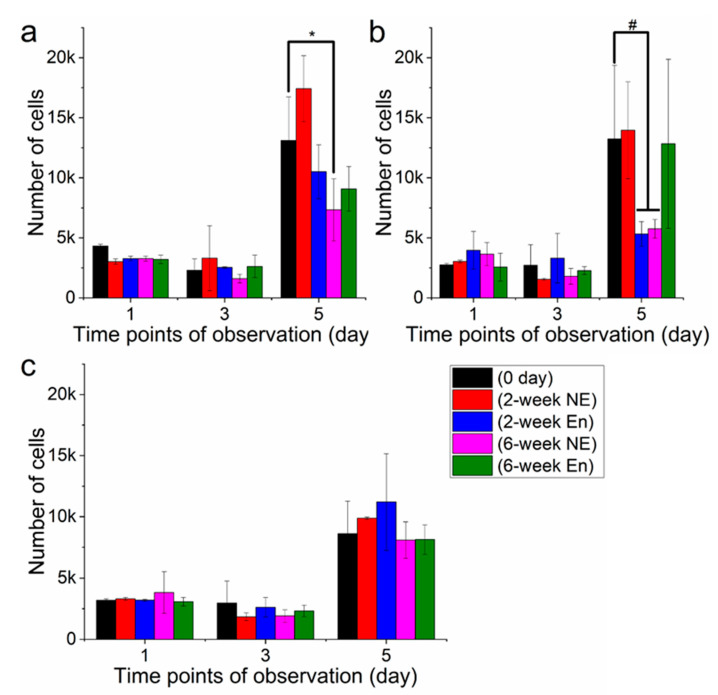
Cell proliferation on degraded films of PHB (**a**), PLA (**b**) and PHB/PLA blend (**c**) *—*p* < 0.05; #—*p* < 0.05.

To assess the interaction between degraded films and 3T3 fibroblasts, the cells were seeded on degraded films followed by a viability test (Figure 10). The UV absorption value is proportional to cell viability, and higher absorption indicates better cell viability and proliferation. The results revealed that films experiencing different degradation durations had distinct cell viability values. For all three different polymer films, there was no significant difference in cell viability between 1st day and 3rd day incubation (Figure 10a–c). It can also be noted that, within the same type of polymer, films after 2 weeks’ degradation demonstrated similar values to the films after 6 weeks degradation in spite of degradation type. However, cell viability varied drastically after 5d’s incubation, which exhibited both in degradation and polymer types. For PHB (Figure 10a), films after 2 weeks’ non-enzymatic degradation had the highest value of cell proliferation, while films after 6 weeks’ degradation showed lower values of cell viability. In comparison to 1 day and 3 days incubation, cell viability value after 5 days had encountered a drastic increase. 

PLA films showed a similar pattern to PHB after 5d’s incubation (Figure 10b). A high level of cell proliferation (around 0.4 × 10^4^) was observed on fresh-made films, films after 2 weeks non-enzymatic degradation and films after 6 weeks’ enzymatic degradation. Unlike PLA and PHB, the blend after 5 days incubation exhibited a different pattern of cell viability with a relatively consistent value around 8 × 10^4^, and the highest value was observed on films after 2 weeks’ enzymatic degradation (Figure 10c). It should also be noted that cell proliferation remained relatively consistent within the same time points regardless of degradation durations and types of films.

### 3.5. Analysis of Surface Structure and Cell Distribution

**Figure 11:**
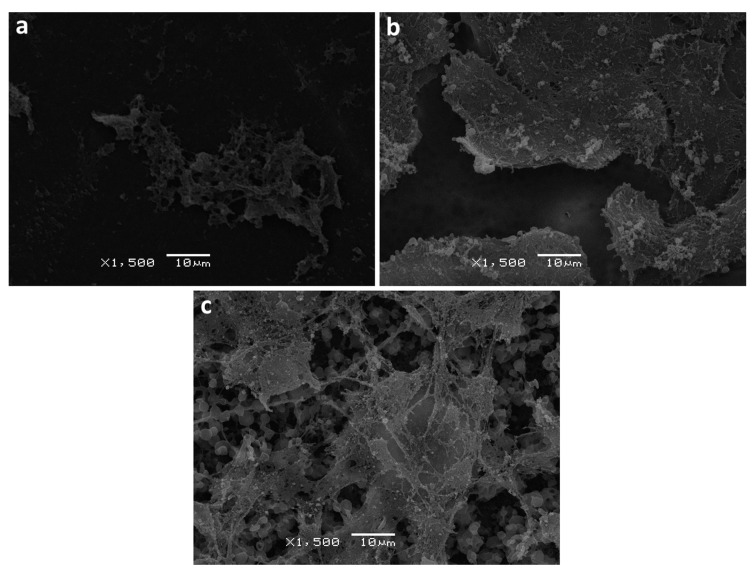
Surface analysis of PHB (**a**), PLA (**b**) and PHB/PLA blend (**c**) after 1-week 3T3 fibroblast cultivation on them (SEM, ×1500).

Scanning electron and fluorescence microscopy methods were utilized in order to further assess the changes in the structure of the film surface as well as cell distribution. Upon film degradation, surface erosion was observed, where the extent of the erosion varied among the polymer types. The samples used for SEM analysis were obtained from the cell experiment after one week’s incubation. As shown in Figure 11, both PHB/PLA blend and PLA exhibited certain degree of surface erosion characterized by disruption of surface morphology, while the surface of PHB remained relatively smooth and intact. It can also be noted that the blend does not possess a homogeneous structure, where it contains round spherical structures. Cell proliferation on PLA and PHB/PLA blend film surfaces was observed to have a broad and relatively even distribution. Unlike PLA, cell distribution displayed a distinct pattern on PHB and PHB/PLA blend surface, which consists of isolated colonies with different sizes.

**Figure 12:**
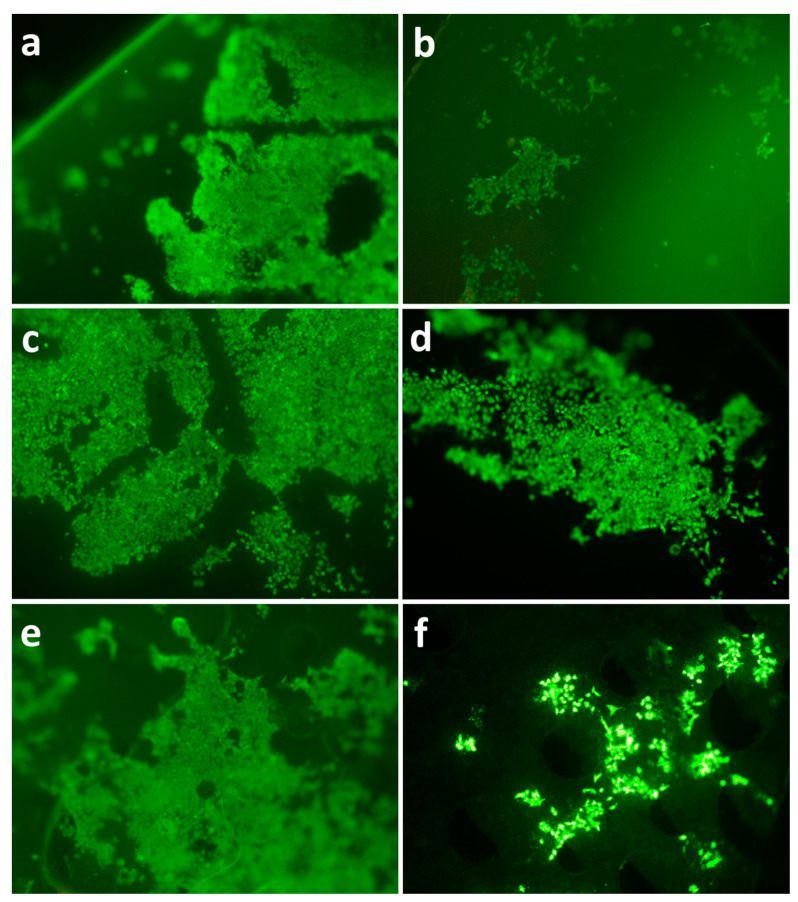
Cell proliferation at 5th day on degraded films from PHB (**a**,**b**), PLA (**c**,**d**), PHB/PLA blend (**e**,**f**); fresh films (**a**,**c**,**e**) and 6-weeks after enzymatic degradation (**b**,**d**,**f**).

Polymer films undergo properties change as the degradation proceeds, which may influence the viability and distribution of seeded cells. Fluorescence microscopy was used to study cell viability and distribution on both fresh and degraded polymer films. The results revealed that 3T3 cells proliferated on all polymer films after 5 days’ incubation. On fresh films the surfaces were covered by a consecutive and dense layer of 3T3 fibroblasts (Figure 12). However, cell distribution on degraded films (42d’s enzymatic degradation) displayed a distinctive pattern compared to fresh films, which is characterized by isolated colonies of different sizes. It can be noted that degraded PLA films had a relatively even cell distribution. Interestingly, there were small holes scattered across degraded PHB/PLA blend surface.

One of the indicators of polymers use in drug delivery and tissue engineering is the analysis of the ability of the polymer surface to adsorb proteins. The degree of BSA adsorption ability was evaluated on the PHB, PLA, and PHB/PLA blend films. The polymer films incubated in PBS were observed to have a different protein adsorption, compared to the films incubated in a lipase solution (Figures S3 and S4, Supplemental Materials). Thus, 1 week films incubation in a PBS solution resulted in a decrease in the amounts of adsorbed protein. Subsequently, it resulted in increased amounts of adsorbed proteins. However, when the films were incubated in a lipase solution, an opposite trend was observed. After the first week, the amount of protein on the surface of the films initially increased and shortly after that the protein concentration dropped. The experiment and its results are described in more detail in the Supplementary Materials (Supplementary Materials Figure S2).

## 4. Discussion

Weight loss is one of the most conspicuous features observed in polymer degradation, indicating the polymer destruction. Figure 2 reveals that PLA has a greater weight loss than PHB, which is in accordance with literature. It is connected with that PHB has high crystallinity [28,37]. Furthermore, weight loss of PHB has no significant differences between its enzymatic and non-enzymatic degradation. 

Molecular weight loss serves as important indication of polymer degradation. Figure 3 reveals that enzymatic degradation induced a greater molecular weight loss than non-enzymatic degradation, which can be explained by involvement of lipase in accelerating the degradation rate. Researches also confirmed that enzymes, like lipase and depolymerase, are more efficient in contributing to the breakdown of polymer chains [38,39]. Although PLA had a smaller initial molecular weight value than PHB, it exhibited a higher loss value (54%) after 6 weeks of enzymatic degradation, where according to the literature, PLA is more prone to degradation than PHB [26,28]. 

The kinetic model parameter (k_D_) demonstrated interesting data. During PHB and PLA homopolymer decomposition in a lipase solution, the kinetics curves had two distinct stages of molecular weight change. At the first stage (the first 2 weeks of degradation) the rate of degradation was twice as high as during the following 4 weeks. This can be explained by the presence of an amorphous component in semicrystalline polymers. It was shown earlier that the amorphous component decomposes much faster than the crystalline one [40,41]. Autocatalysis is observed during the decomposition of PLA in a phosphate buffer (Table 2). The absence of this phenomenon during the enzymatic decomposition of PLA is explained by the higher rate of decomposition, at which autocatalysis is not observable. The static state of PHB between 4 and 6 week’s degradation might result from the impediment imposed by the crystallized inner part, which was exposed to the buffer after depletion of amorphous outer regions. It was suggested that further crystallinity analysis may help to explain the unchanged state. 

Thermal properties, such as crystallinity, serve as an important indicator of polymer degradation, where the properties changes vary among different polymers and copolymers [28]. It was reported that the rate of erosion of melt-crystallized films significantly decreased as the degree of crystallinity increased [39]. DSC analysis revealed (Table 2) that PHB remained at a constant melting temperature both in blend and pure PHB, which was also in accordance with literature [42]. The crystallinity of pure PHB displayed relatively small change after 6 weeks’ degradation, suggesting that PHB films still maintained a relatively ordered crystalline structure, which further suggests that PHB had the lowest weight loss and degradation rate compared with the blend and PLA (Figure 7). Pure PLA exhibited reduced crystallinity after 6 weeks’ degradation, which may indicate the erosion of crystalline phase.

Previous research also confirms that enzymes came into effect when the ordered structures of PHB were dismantled by initial degradation [43,44]. The slow degradation of PHB indicates that high polymer crystallinity restricts the effectiveness of enzymes, which slows down the degradation process. Enzyme cannot penetrate highly ordered structures of PHB films at this stage but can penetrate the amorphous phase of PHB and hydrolyze polymer chains. The high molecular weight loss of PHB is associated with cleavage of PHB chains in amorphous phase, which was earlier demonstrated by the work of Zhuikov et al. [45]. However, this effect of less susceptibility to degradation by enzymes at a later stage is also present for PLA films, whereas the rate of its degradation is much higher (Figure 6b).

The blend fabricated through mixing PHB and PLA was expected to demonstrate weight loss that would be an average of the value of its primary components PHB and PLA. However, the experimental results showed that blend underwent the highest weight loss after 6 weeks’ enzymatic degradation. It was observed that the PHB/PLA films had a pronounced roughness and porosity after 6 weeks’ enzymatic degradation, which supports the fact that the blend films degraded faster than PLA films following 6 weeks of enzymatic degradation. The unexpected data were obtained upon analysis of the change in the PHB/PLA blend molecular weight. The blending of PHB with PLA reduced the degradation rate and, turning out to have the smallest rate under both enzymatic and hydrolytic degradation. Unlike k_D_ of PHB and PLA homopolymers, the k_D_ of PHB/PLA blends stayed the same during both during enzymatic degradation and during lipase-free hydrolysis (Table 1). The curve had one relatively straight section (Figure 6c). This behavior can be attributed to a possible interaction between PHB and PLA within the mixture. The degradation rate of the blend is about 4-times less than the rate of the individual homopolymers at the first stage. Notably, the crystallinity degree of the polymers in the blend was also observed to be lower (Figure 2). The blend’s k_D_ value is close to the PLA k_D_ value at the second stage of its degradation, which suggests that PLA could contribute to the blend’s degradation at least during the late stages of the course of the experiment (3–6 weeks). The analysis of blend degradation curves showed that the correlation coefficients of both nonautocatalic and autocatalic models were quite close (Table 2), which suggests that the contribution of autocatalysis cannot be excluded. Thus, regarding the curve of PHB/PLA blend weight loss during its enzymatic degradation (Figure 1), we can assume that this effect may be due to the removal of low-molecular degradation products, whereas they are retained in the polymer matrices of PHB and PLA. This can lead to a more uniform and a less pronounced degradation process in the blend.

PHB/PLA blend possessed a more complicated pattern with an increase in crystallinity in PHB component, which may have signified a temporal positive impact of the degradation stages of the initial erosion of amorphous phases on the crystallinity. According to the DSC curve (Figure 5), the PHB and PLA polymers were immiscible. The characteristic peaks are well distinguishable for each of the polymers. The glass transition temperature and the melting temperature for PLA were found to be 48.8 °C and 146.6 °C, respectively. Moreover, the peak of PLA crystallization (95.1 °C) was observed. This peak is not always visually accessible upon use of polymer mixtures [26]. The typical melting point peak of PHB was at 175.4 °C, and in contrast to the PLA, the crystallization temperature via cooling was at 70.9 degrees. It was shown also that PLA and PHB are immiscible. The blending of these polymers may compromise the ordered solid structures of polymers, making the blend vulnerable to the enzymatic attack [26,46].

These data indicate that the film represents inclusions from PHB and PLA, located randomly throughout the volume. The PLA component in blend had lower crystallinity in enzymatic degradation than that in non-enzymatic degradation, which accounts for highest weight loss of the blend upon enzymatic degradation. It is worth noting that in comparison to pure polymers, both PLA and PHB components in blend experienced a decrease in crystallinity, which is related to the immiscibility and structure disruption between PLA and PHB. In this research, we speculated that following initial erosion of ordered regions, enzymes were capable of penetrating inside the regions with greater fragility and immiscibility that were formed by combination of PHB and PLA, therefore accelerating the degradation rate. Upon suggesting that the polymers are in fact immiscible, this implies a potential increase in the polymer molecules’ surface area, which furthermore may elevate the degradation rate. Surprisingly, this immiscible nature of PHB/PLA blend leads to enzymatic degradation with uniform rate, since enzymes better penetrate into the unorganized polymer blend matrix, especially at the late stage of degradation process, in comparison with the pure polymers, their components: PHB and PLA. 

Analysis of the particulate degradation products from polymer films after first week showed very low concentrations of the released particles. The highest concentration was achieved only during PHB films degradation, which was in agreement with our results. This explains the largest decrease in PHB molecular weight in the first week of degradation. After the second week of incubation in water, it became possible to detect nanoparticles. The highest concentrations correspond to particles with a size of 340 nm for PHB, 300 nm for PLA, and 340–530 nm for a composite. The particles of about 200 nm were discovered after the first week of PHB incubation. During this time polymers of this type not have enough time to undergo the decomposition process due to their hydrophobicity. Therefore, the most accurate explanation for this observation is the washing out of oligomers and unbound polymer from the bulk of the product. During the two weeks of incubation, which in turn elevates the particle fraction and leads to the formation of the particle peaks of the order of 10 nm. Particles with the size over 5500 nm are considered an artifact, arising from an insufficient centrifugation, as well as the initial fraction of the polymer, which gives rise to smaller nanoparticles.

Films seeded with cells may have different changes in properties compared to pure films. The molecular weight analysis showed that the growth of 3T3 cells on PLA and blend helped to accelerate the degradation rate, which may have been influenced by the cells’ metabolism. Moreover, 3T3 cells had a wider distribution across PLA’s and the blend’s surface (confirmed by images obtained from SEM and fluorescence microscope). The PHB degradation has shown an opposite trend, where no significant difference in cell experiment and degradation experiment, which was confirmed by a relatively unaffected PHB surface. It was suggested that the highly ordered structure of PHB restricts the possible effects imposed by cell metabolism in the initial stage of degradation. Besides, it can also depend on the overall number of cells grown on films at each time point, which might require further investigation to reveal the distinctive pattern of PHB.

It can be observed that PLA had greater molecular weight loss in cell experiment than in non-enzymatic degradation, while molecular weight loss of PHB and PHB/PLA blend in non-enzymatic degradation was higher than in cell experiment (Table 4). 

The molecular weight loss of enzymatic experiment was higher than cell experiment. A possible explanation for this effect is that upon enzymatic decomposition, the enzyme is provided with a large polymer area, which leads to an accelerated degradation. Cells also accelerate the degradation of polymers but act locally. In contrast to cell-based components, a higher PHB degradation rate in the non-cell (PBS) can be explained by the facilitated leakage of the solutions into the polymer matrix. Cells spread out on the surface of the polymer and, and show a smaller effect on it due to the hydrophobicity of the polymer. In cell investigation PHB/PLA blend also demonstrated more uniform and slower degradation process than PHB and PLA that can be explained by the uneven ultrastructure of the polymer blend matrix and, therefore, its better availability for cleavage by cellular enzymes. 

Cell viability test is widely used for evaluation of cell proliferation on polymer films [35,47]. Higher cell viability value indicates better cell proliferation and adhesion. The blend and PLA had higher cell viability values after 2 weeks incubation, which implies better cell adhesion and proliferation (Figure 9). It is also suggested that PLA and blend films display better interaction with 3T3 cells, which is confirmed by images obtained from SEM. This difference in cell viability between polymers may be related with a combination of favorable properties of polymer film surface, such as hydrophilicity and microstructure, which requires further investigation. 

Cell experiment on degraded films revealed that there is no significant difference in cell viability between 1d and 3d’s incubation, which may be explained by a small size of films and insufficient incubation time. After 5d’s incubation, it was observed that films with different durations of degradation exhibited distinctive cell viability values. For PHB, fresh films and films after 2 weeks’ degradation had higher cell viability than films with 6 weeks’ degradation, indicating that the degradation duration may have shown a negative correlation with cell proliferation. PLA had a similar cell viability pattern as PHB after 5d’s incubation the only exception with the films undergoing a 6 week enzymatic degradation, which showed higher cell viability. It was suggested that there is no linear correlation between degradation duration and cell viability. Interestingly, the blend displayed relatively stable cell viability in spite of films with different degree of degradation, which indicates that the biocompatability of PHB/PLA blend was retained after 6 week degradation. This effect is also correspondent with more uniform degradation process of the blend.

It was reported in literature that the surface structure of polymers can influence proliferation and adhesion of cells and also that it it depends on specific microstructures of certain biopolymers [18,47]. Images obtained from scanning electron microscope revealed that the blend had higher degree of surface erosion (Figure 11c), which can be explained by the fact that the polymer blending disrupted the ordered structure of pure PLA and PHB, making the blend prone to degradation. PHB retained its relatively smooth and intact surface, which can be attributed to its high crystallinity and high viscosity. The difference in cell distribution may be related to the polymer’s initial surface properties, which influenced cell distribution pattern during the experiment. Meanwhile, it also could be a result of interaction between cells and polymers, which impose a mutual effect on both degradation course and cell proliferation.

The micro- and nanoparticles’ analyses during the degradation of polymers in milli-Q water showed presence of polymer particles in solution with the following size distributions: PHB: 3, 10, 340 nm. PLA: 0.9, 300 nm and for PHB\PLA: 4.1, 340–530 nm. Particle sizes of 300–530 nm can correspond to polymer particles that are not bound to the polymer matrix and released from the bulk of the polymer film. Nanoparticles with size range of 0.9 to 4.1 nm were also observed and were suggested to be a decomposed part of the polymer.

According to the results (Figure S5), cell proliferation on centrifuged samples did not differ from cells in the control medium, which suggests that the products of PHB degradation are not toxic for them, whereas non-centrifuged PHB powder dispersion in medium inhibited cell proliferation. However some authors implied that PHB nanoparticles do have a toxic effect [33].

The fluorescence microscopy data shows that cells exhibited better proliferation on fresh made films after 5d’s incubation, which indicates that all polymer films were biocompatible. It was also observed that degraded films of blend and PHB had different cell distribution patterns compared to PLA, which were characterized by a combination of large and small colonies. We speculated that this difference may be related to surface structure and molecular weight. The blend suffered severe surface erosion after 6 weeks’ degradation, which disrupted and restricted cell proliferation to isolated areas with different scales. Although PHB did not experience a high degree of surface erosion after 6 weeks’ degradation, it experienced the highest molecular weight loss, which could be a factor causing the scattered pattern of cell distribution.

Surprisingly, the uneven distribution of cells on polymer films can also be the reason for their slower and more uniform degradation. It was suggested also that the information about cell distribution patterns can be of great benefits for designing 3D-scaffolds seeded with cells for tissue and organ repair. 

In bacterial cells, PHB is accumulated in special granules—carbonosomes. It was shown that PHB in such granules is at amorphous condition, which is maintained by a series of PHB-binding proteins [48]. Such an amorphous conformation of PHB allows the bacterial cell to use this biopolymer as a carbon source for its vital functions. The blending of PHB with PLA can be, with some reservations, a prospective tool to mimic a natural irregular conformation of PHB in bacterial carbonosomes. This approach can help to achieve the desired kinetics of polymer biodegradation for its application in regenerative medicine and tissue engineering.

## 5. Conclusions

It was shown that PLA films lost the mass and molecular weight faster than PHB, which was consistent with the literature data [26,28]. In both polymers there was a two-stage pattern in the change of the enzymatic degradation rate and a one-stage pattern for non-enzymatic degradation rate. In the PHB/PLA blend the aforementioned pattern was not observed: the blend underwent a slower and more uniform one-stage process of enzymatic degradation. Enzymatic degradation significantly accelerated the degradation rate of all polymers compared to non-enzymatic hydrolytic degradation. The PHB degraded slower, which indicates its greater stability. According to the DSC results, PHB and PLA did not mix during the manufacture of the composite. We propose that the immiscible nature of PHB/PLA blend can be the reason for its greater stability during enzymatic degradation, due to possible better enzymes penetration into the unorganized polymer matrix of the blend, whereas the highly ordered structure of PHB and PLA severely limited enzyme penetration.

Studies of the effect of cell growth on the film surface showed that the viability of MSCs growing on PHB/PLA was not influenced at the time of enzymatic and non-enzymatic degradation of polymer films, while there were large differences in cell growth on both PHB and PLA films at different durations. The blend and PLA films exhibited a certain degree of surface erosion characterized by disruption of surface morphology, while the surface of PHB remained relatively smooth and intact. It was associated with the uneven distribution of cells (in form of large and small colonies) on polymer film surface. In cell growth studies PHB/PLA blend also demonstrated a more uniform degradation process with lower rate than its polymer components, PHB and PLA. We propose that it also can be linked to the unorganized structure of the blend’s polymer matrix.

The size of particles released from polymer films can be divided into two groups: nanoparticles (0.9 to 4 nm) and submicroparticles (300–530 nm). The detection of these particles confirms the hypothesis about the release of oligomers and unbound polymer fragments from the bulk of polymer film, leading to a significant mass reduction of the films and MM at the initial stages of polymer degradation. It is possible that the polymeric biodegradation products can have an additional effect on growth of MSC and 3T3 fibroblasts upon their culture on polymer films.

As a result, the irregular and imperfect structure of the matrix of biodegradable polymer blends is not a limiting factor for their biomedical use. It is not necessary to always manufacture perfectly mixed polymer composites to achieve their biodegradation with appropriate kinetics. Nevertheless, novel techniques should be developed that will allow scientists to control the irregular polymer structure. Moreover, it can be a biomimetic approach for designing a natural irregular conformation of PHB to achieve the controlled kinetics of polymer biodegradation in human tissues. It allows to create e.g., biodegradable scaffolds with desired properties for regenerative medicine and tissue engineering.

## Figures and Tables

**Table 1:** Kinetic model parameters and coefficients of determinations (R^2^) for the degradation of PHB, PLA and PHB/PLA blend in phosphate buffer without lipase and phosphate buffer with lipase.

Sample	PBS		Lipase	
	k_D_ (Week^−1^)	R^2^	k_D_ (Week^−1^)	R^2^
PHB	0.12 ± 0.01	0.96	0.28 ± 0.046 0.01 ± 0.002 *	0.97 0.98
PLA	0.1 ± 0.009	0.97	0.26 ± 0.035 0.07 ± 0.02 *	0.98 0.93
PHB/PLA	0.07 ± 0.01	0.97	0.07 ± 0.007	0.96

* 2nd sections of the curve with different rates of degradation. Explanations in the text.

**Table 2:** Assessment of the contribution of the autocatalysis process to the decomposition rate of PHB, PLA and PHB/PLA blend films.

Degradation Model	Polymer Sample	R^2^ Nonautocatalic Model	R^2^ Autocatalic Model
Non-enzymatic	PHB	0.91	0.85
	PLA	0.97	0.97
	PHB/PLA	0.87	0.84
Enzymatic	PHB	0.76	0.61
	PLA	0.90	0.83
	PHB/PLA	0.94	0.91
Cell-cultivating	PHB	0.95	0.93
	PLA	0.85	0.74
	PHB/PLA	0.97	0.98

**Table 3:** DSC parameters of polymer films (degradation duration: 6 weeks).

Polymer	Samples	*T_m_* (PHB), °C	*T_m_* (PLA), °C	*X_c_* (PHB), %	*X_c_* (PLA), %
PHB	Fresh	176.5	---	63.0	---
	NE	175.0	-	58.6	-
	En	176.1	-	64.0	-
PLA	Fresh	-	148.1	-	34.0
	NE	-	153.4	-	29.2
	En	-	147.0	-	29.5
PHB/PLA	Fresh	175.4	146.6	46.7	18.5
	NE	175.2	151.3	52.1	21.5
	En	174.8	147.0	66.3	13.2

**Table 4:** The comparison of molecular weight loss after 2 weeks in the non-enzymatic, enzymatic, and cell growth degradation models.

Polymer Films	Molecular Weight Loss, %		
	Enzymatic Degradation	Non-Enzymatic Degradation	Cell Experiment
PHB	45 ± 2.2	27 ± 1.3	21 ± 1
PLA	42 ± 2.1	25 ± 1.2	33 ± 1.6
PHB/PLA	23 ± 1.2	14 ± 0.7	10 ± 0.5

## Data Availability

Data sharing not applicable.

## Supplementary Materials

The following are available online at https://www.mdpi.com/2073-4360/13/1/108/s1, Figure S1. The viscosity loss of polymer films during their in vitro degradation. Figure S2. The viscosity loss of polymer films during cell growth on them. Figure S3. BSA absorption on PHB, PLA, and PHB/PLA blend films (after non-enzymatic degradation). Figure S4. BSA absorption on PHB, PLA, and PHB/PLA blend films (after enzymatic degradation). Figure S5. Evaluation of MSC proliferation in the media containing particulate degradation products of PHB by XTT on the 1st, 5th and 7th days of cell incubation. Data presented as mean ± SD, *—*p* < 0.05 (vs. Powder group). Figure S6. Surface analysis of after 1-week 3T3 fibroblast cultivation on PHB/PLA blend film. Figure S7. Surface analysis of after 1-week 3T3 fibroblast cultivation on PLA film. Table S1. The comparison of viscosity loss after 2 weeks of cell growth on PHB, PLA, and PHB/PLA films.
