# Supplementary material for: The Growth of 3T3 Fibroblasts on PHB, PLA and PHB/PLA Blend Films at Different Stages of Their Biodegradation In Vitro
Source: Polymers (Basel). 2020 Dec 29;13(1):108. doi: 10.3390/polym13010108 (PMC7795568; doi:10.3390/polym13010108)
Supplement: Supplementary file 1 [file polymers-13-00108-s001.pdf]

Supplementary data

S1. Viscosity loss of biopolymer films

Viscosity is one of the key properties of biopolymers, the decrease of which indicates the ongoing degradation process and its mechanism. Meanwhile, the change of viscosity also represents the change of molecular weight that is associated with biopolymer degradation. It can be observed that the viscosity decreased for all three biopolymers during both enzymatic and non-enzymatic degradation (Fig. 1). Compared with non-enzymatic degradation, all three polymers have larger viscosity loss with the involvement of lipase. Moreover, PLA has the highest viscosity loss 45% under enzymatic degradation, whereas PLA: 41%, and blend: 36%. We can also notice that PHB has the highest initial viscosity, while PLA and blend have smaller values. Although the viscosity of PHB had a drastic decrease during the first 4 weeks, it remained nearly unchanged in the last two weeks' degradation.

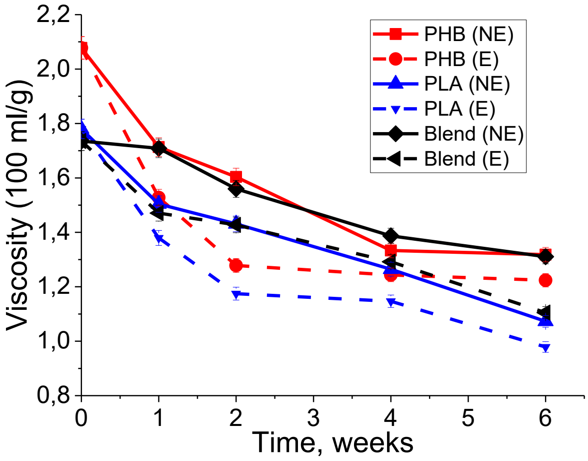

Figure S1. The viscosity loss of polymer films during their in vitro degradation.

To investigate how viscosity of polymers might change with the seeding of 3T3 cells, we also performed measurement in cell experiment. As shown in Fig. 2, the viscosities of all polymers decreased during cell experiment, and PLA possessed the largest viscosity loss about 30% (PHB: 24%, blend 19%).

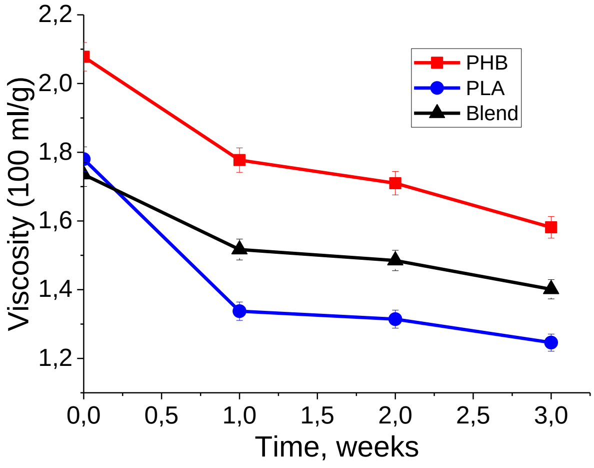

Figure S2. The viscosity loss of polymer films during cell growth on them.

The comparison of viscosity loss between degradation experiment and cell experiment after 2 weeks is presented in Tab. S1.

Table S1. The comparison of viscosity loss after 2 weeks of cell growth on PHB, PLA, and PHB/PLA films.

Viscosity loss %	Enzymatic degradation	Non-enzymatic degradation	Cell experiment (2 weeks)

	(2 weeks)	(2 weeks)	
PHB	39	30	23
PLA	35	24	30
PHB/PLA	21	16	19

We can observe that PLA and Blend had greater viscosity loss in cell experiment than that in non-enzymatic degradation, while viscosity loss of PHB in non-enzymatic degradation is higher than in cell experiment. Besides, the viscosity loss of enzymatic experiment is higher than cell experiment. A possible explanation for this effect is that in the case of enzymatic decomposition, the enzyme is provided with a large polymer area, which leads to an acceleration of degradation. Cells also accelerate the degradation of polymers but act locally. Faster degradation of PHB in a phosphate buffer compared to cells may result from the penetration of solutions into the volume of the polymer film. Cells are spread on the surface of the polymer and, due to the hydrophobicity of the polymer, have a lesser effect on it.

S2. Surface protein absorption

The surface absorption ability is one of the essential characters of biopolymers. To investigate the effect of degradation on surface absorption ability, we performed protein absorption test on both fresh and degraded films. As shown in Fig. 3, non-enzymatically degraded films displayed distinctive abilities of surface protein absorption during different observation points. After one week's degradation in phosphate buffer, BSA absorption decreased for all polymer films, and films with higher portion of PLA suffered lower decrease of BSA absorption. Furthermore, the absorption ability of all polymer films encountered different degrees of increase after two weeks' degradation. For the last two weeks, films with high proportion of PLA had another decrease of BSA absorption, while the absorption ability of PHB and PHB/PLA (75/25) continued to increase. It is worth noting that PHB/PLA (50/50) possessed the highest protein absorption after 4 weeks' degradation in phosphate buffer.

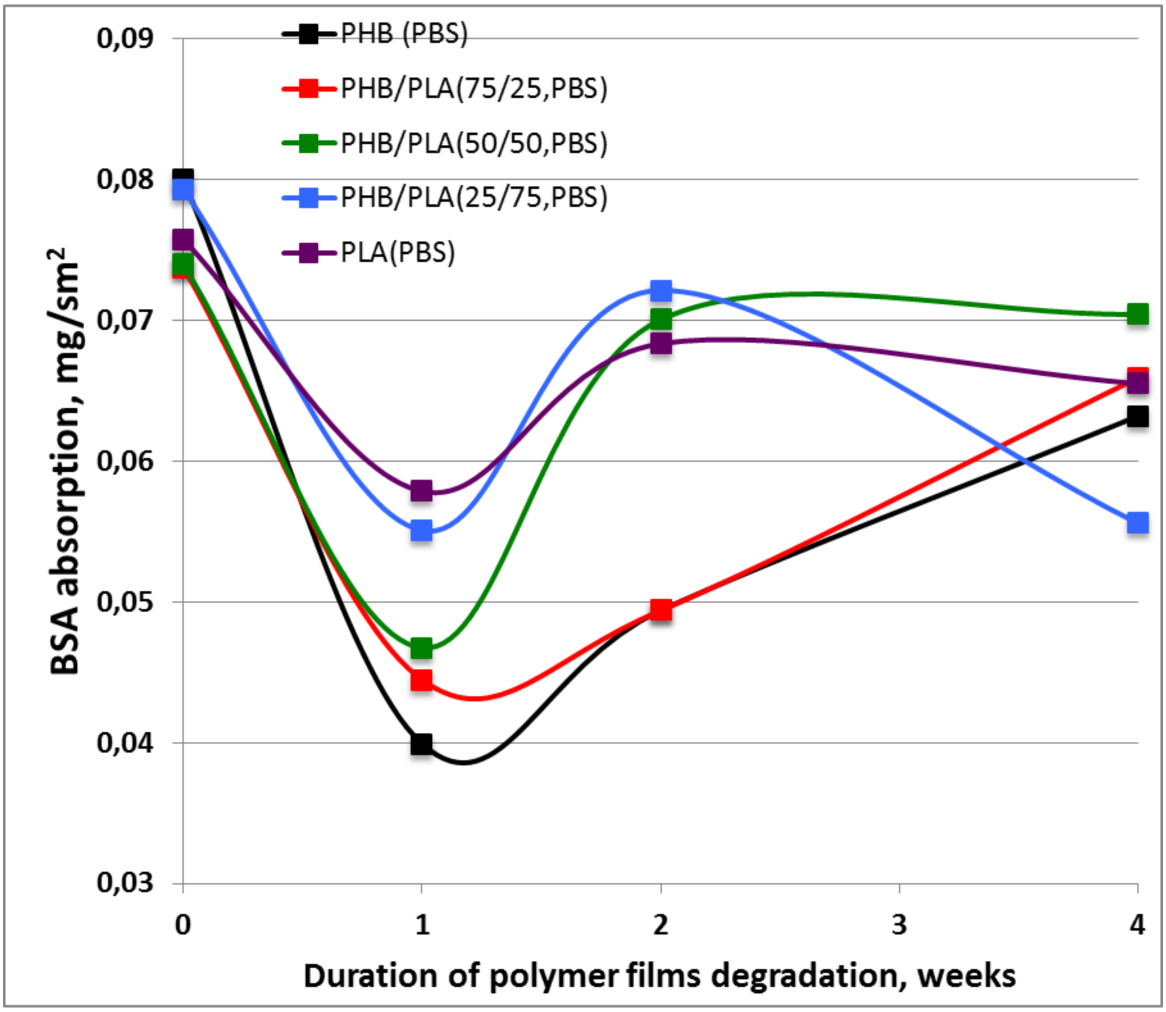

Figure S3. BSA absorption on PHB, PLA, and PHB/PLA blend films (after non-enzymatic degradation).

Unlike films that degraded in phosphate buffer, enzymatically degraded films exhibited a different pattern concerning the changes of absorption ability. All polymer films showed an increase of protein absorption after one week's degradation, except for PHB/PLA (25/75) which had a linear decline of absorption during the whole degradation course (Fig. 4). During the subsequent 3 weeks, all polymer films encountered a continuous decrease of protein absorption. It should be noticed that PHB/PLA (50/50) remained the highest level of BSA absorption, while PHB suffered the greatest loss of absorption ability.

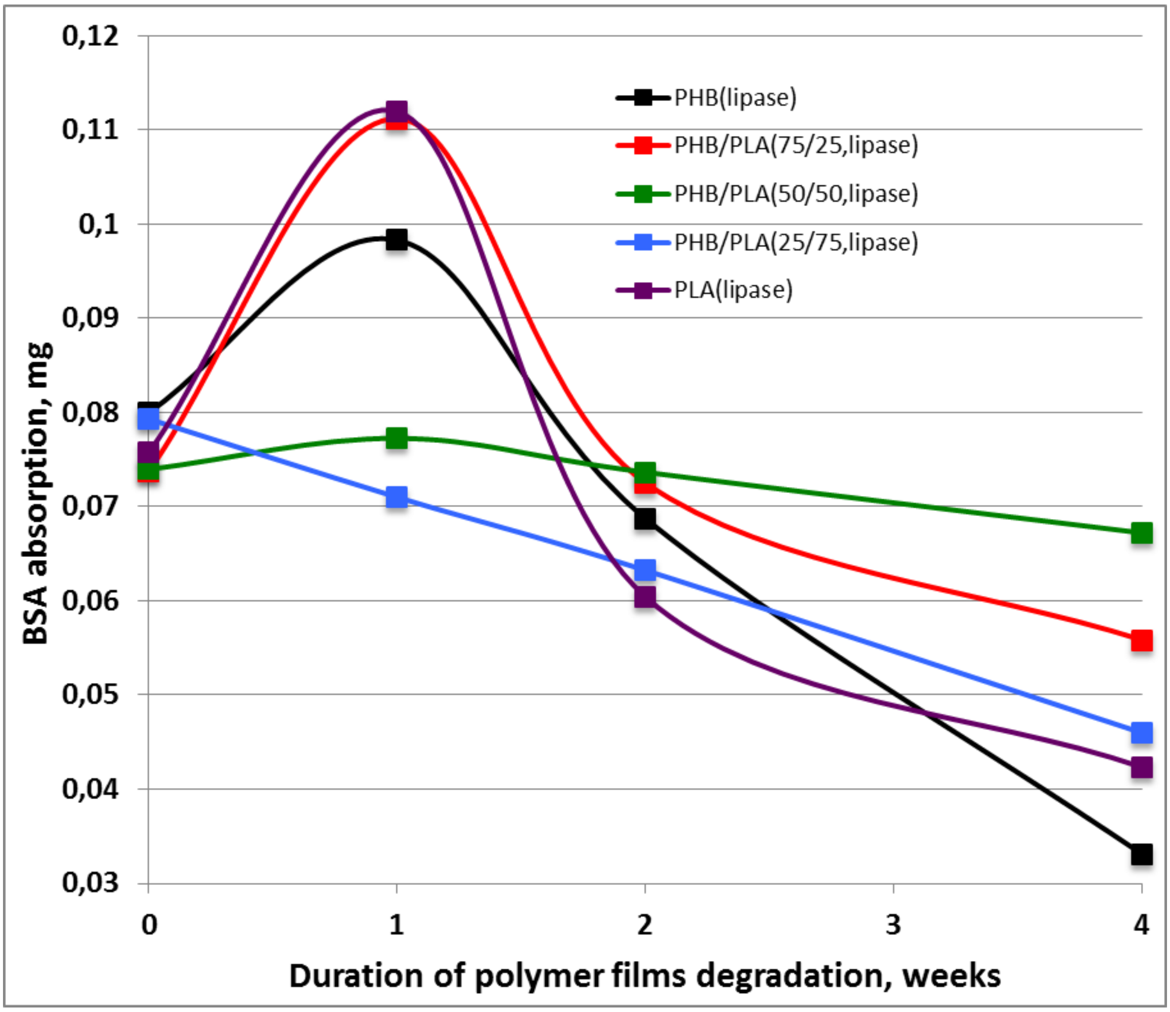

Figure S4. BSA absorption on PHB, PLA, and PHB/PLA blend films (after enzymatic degradation)

S3. Study of mesenchymal stem cells cultivation

S3.1 Method of mesenchymal stem cells isolation and cultivation

Mesenchymal stem cells (MSC) isolated from adipose tissue of newborn rat pups were grown in the presence of the obtained samples of centrifuged solutions supplemented with 10% fetal calf serum (FBS) (Biological Industries, Israel) in Medical Faculty of RUDN University (Moscow) and kindly provided by Assoc. Prof. Muraev A.A. [34]. MSCs cultured in a standard growth medium for 1, 5, 7 days and microparticles (powder) from PHB were used as controls. To study MSC proliferation XTT cell proliferation test was used as for study of NIH 3T3 fibroblasts proliferation (see section 2.10).

S3.2 Study the effect of PHB degradation products on the MSC growth

To check the possible effect of the decomposition products of PHB on the cell growth investigation of their effect on growth of MSC was carried out (Fig. S5).

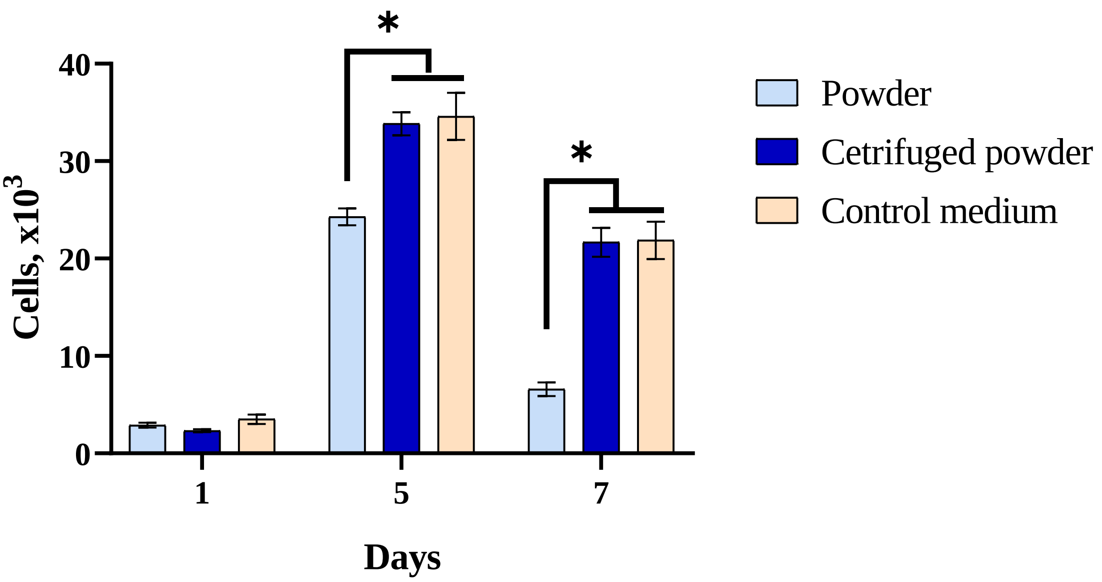

Figure S5. Evaluation of MSC proliferation in the media containing particulate degradation products of PHB by XTT on the 1st, 5th and 7th days of cell incubation. Data presented as mean ± SD, * -p < 0,05 (vs. Powder group).

S4. SEM images of cells grown on the polymer films

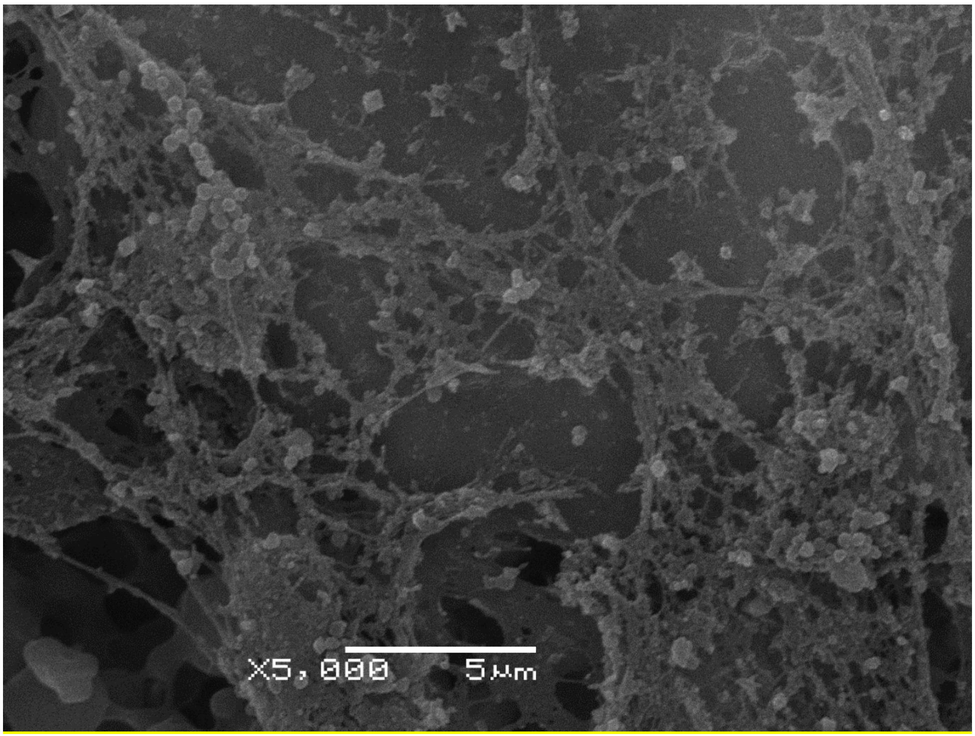

Figure S6. Surface analysis of after 1-week 3T3 fibroblast cultivation on PHB/PLA blend film.

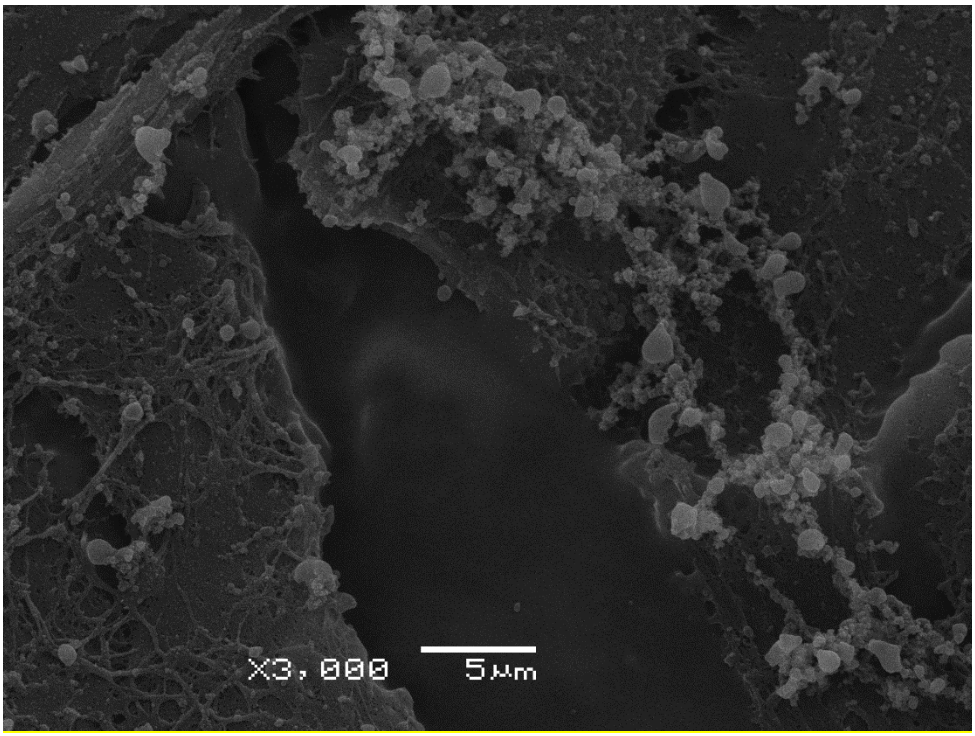

Figure S7. Surface analysis of after 1-week 3T3 fibroblast cultivation on PLA film.
